# Antimicrobials Used in Backyard and Commercial Poultry and Swine Farms in the Philippines: A Qualitative Pilot Study

**DOI:** 10.3389/fvets.2020.00329

**Published:** 2020-07-08

**Authors:** Toni Rose M. Barroga, Reildrin G. Morales, Carolyn C. Benigno, Samuel Joseph M. Castro, Mardi M. Caniban, Maria Fe B. Cabullo, Agnes Agunos, Katinka de Balogh, Alejandro Dorado-Garcia

**Affiliations:** ^1^Food and Agriculture Organization of the United Nations—Philippine Component on the Global Efforts to Combat Antimicrobial Resistance Using One Health Approach (GCP/GLO/UK/710), Quezon City, Philippines; ^2^Department of Agriculture, Bureau of Animal Industry, Quezon City, Philippines; ^3^Department of Agriculture, National Meat Inspection Service, Quezon City, Philippines; ^4^Food and Agriculture Organization of the United Nations Regional Office of Asia and the Pacific, Bangkok, Thailand; ^5^Food and Agriculture Organization of the United Nations, Rome, Italy

**Keywords:** farm level, antimicrobials, surveillance, poultry, swine, Philippines, LMIC

## Abstract

Chicken and pork are the most frequently consumed meat products in the Philippines. Swine and poultry are reared in either commercial farms (CMf) or backyard farms (BYf); the latter production system is relatively common and essential to food security in low- and middle-income countries (LMICs) such as the Philippines. Similar to resource-limited LMICs, antimicrobial use (AMU) surveillance has not yet been established; thus, AMU in food animals is a knowledge gap in understanding the emergence of antimicrobial resistance (AMR) in zoonotic foodborne bacteria in the country. This qualitative AMU pilot study aims to describe the antimicrobial active ingredients (AAIs) used and associated AMU practices (e.g., source of AAIs and informed AMU decisions) by poultry and swine CMf and BYf in the Philippines. Ninety-three farms across four regions in the Philippines voluntarily provided AMU information as part of a larger biosecurity and good practices study. The percentage of farms using AAI over the total number of farms was the metric used to describe AMU. In total, there were 30 AAIs used (CMf: *n* =27 and BYf: *n* = 13); per farm, the number of AAIs used ranged from 1 to 7. The spectrum of AAIs was more diverse in swine (*n* = 24) compared to poultry (*n* = 18). Enrofloxacin was the most frequently reported AAI in poultry (33%) and swine (36%) farms. Respiratory diseases were the most frequently reported reason for AMU in both species. Between production systems, significant differences were observed in the percentage of farms using amoxicillin (27% CMf vs. 3% BYf), colistin (17% CMf vs. 3% BYf), and oxytetracycline (12% CMf vs. 39% BYf). In terms of AMU practices, of important concern was the over-the-counter access of AAIs at retail outlets and the limited veterinary oversight in BYf. Our data indicated that antimicrobials critically important for human medicine are frequently used in poultry and swine farms in the Philippines. This study can inform the development of guidelines for curbing AMR through prudent AMU and serves as a reference point for AMU surveillance capacity development in the Philippines.

## Introduction

Low- and middle-income countries (LMICs) in certain regions of the world, such as Southeast Asia, are disproportionately burdened with enteric foodborne illnesses (1). Resistance to antimicrobials among zoonotic foodborne bacteria poses an additional concern (2). As such, LMICs have received special attention toward the mitigation of the impacts of antimicrobial resistance (AMR). Recent evidence suggests that antimicrobial use (AMU) in food and agriculture sectors is linked to the development of AMR in bacteria (3, 4). Furthermore, temporal correlations between AMR in zoonotic foodborne organisms in both animals and in people have also been reported (5, 6). Understanding AMU and associated practices in major food production sectors is an essential step to developing interventions to reduce the emergence and dissemination of AMR from animals to human populations.

In the Philippines, the agricultural sector contributes to 9% of its national gross domestic product (GDP), and 29% of the labor force is employed in agricultural services. Livestock and poultry production outputs rank second (25%; 27 million tons) next to crops (49%) in the country's total agricultural production. For the past 30 years, the 85% increase in the human population has been accompanied by a 195 and 332% increase in the volume of swine and poultry production, respectively (7). Similar to the Philippines, chicken and pork among the animal-sourced food are the most frequently consumed in Asia and are also implicated in foodborne illnesses (8). Increasing quantities of antimicrobials are expected to be used with the rapid growth of poultry and swine production in LMICs (9), emphasizing that these sectors are a priority for inclusion in AMU/AMR surveillance programs.

Veterinary services in the Philippines have established policies related to the sale, prescription, and distribution of antimicrobial veterinary medicinal products (VMPs) long before AMR became a global public health issue (10). However, similar to other resource-limited countries (11), weakness on implementation of standards for VMPs, lack of strict enforcement on issuing veterinary prescription to farmers, accessibility of farmers to purchase antimicrobial VMPs in local agriculture-veterinary (agrovet) supply/retail outlets, and lack of awareness on the prudent use of antimicrobials may fast-track the occurrence of AMR. Veterinarians employed by agrifood companies/integrators and allied industries such as feed and pharmaceutical industries, and diagnostic services/independent consultants provide diverse type of service to the livestock and poultry sectors and have established valid veterinary–client–patient relationships (VCPR), and thus have an important role in animal health and food safety. However, veterinarians servicing food animals in a rural setting such as villages within municipalities and cities are limited. Animal health services are typically provided by a network of regional and provincial veterinarians and paraveterinarians. Paraveterinarians are veterinary paraprofessionals commonly known as livestock inspectors, meat inspectors, and agricultural technicians employed by local government units (LGUs) who are trained by government veterinarians, though not yet recognized by the veterinary statutory body, to reach municipalities/cities that are located in remote areas. They have formal training in animal husbandry and some animal health and AMU dispensing training provided by national or regional veterinary authorities.

Gaps in VMP regulation might contribute to food safety (i.e., drug residues) and AMR-related health risks. Recent studies in the country have documented high prevalence and widespread distribution of bacteria resistant to certain antimicrobials (12–14). Of important public health concern is the detection of *Escherichia coli* harboring extended-spectrum beta lactamase-conferring genes (ESBLs) among swine (57.41%) (12) and poultry (66.67%) (13), and high prevalence of quinolone (nalidixic acid) resistant *Campylobacter* spp. from retail chickens (98.1%) (14) in the Philippines. Similarly, in pork products, a high prevalence of resistance to beta-lactams cefazolin (100%), cefuroxime (100%), and cefoxitin (100%) (15) and multidrug resistant *Salmonella* (15, 16) have been reported. In parallel to these findings, efforts to have a nationwide AMR surveillance have just started in 2018 as part of the Philippines AMR National Action Plan (NAP) (17). However, AMU surveillance in the animal sector in the Philippines is yet to be established. Information on the extent of AMU is an indispensable step to tackle AMR. In other countries with well-established AMU programs, integration of AMU and AMR data across multiple surveillance components and sectors (humans, animals) informs the development of One Health evidence-based policies for AMR and enables the monitoring of interventions (17) whether these are industry- or government-driven (18). Global requirements to submit data on antimicrobials intended for use in animals will therefore enable the evaluation of impacts from various directives to reduce AMU in animals (19–21). Built onto NAPs, refinements of husbandry practices, on-farm biosecurity, and management of bacterial infections are complementary preventive approaches to improve production while reducing the need of antimicrobials (22). An understanding of AMU practices and other drivers for AMR are fundamental for the enhancement of food safety programs in the poultry and swine production continuum.

In the context previously described, this qualitative pilot study aims to describe the antimicrobial active ingredients (AAIs) used and AMU practices (e.g., source of AAIs and informed AMU decisions) by poultry and swine CMf and BYf in the Philippines. This study can inform the development of guidelines for curbing AMR through prudent AMU and serves as a reference point for AMU farm surveillance capacity development in the Philippines.

## Materials and Methods

This pilot study on AMU is part of a larger project delivered by the Food and Agriculture Organization of the United Nations (UN FAO) in the Philippines and globally (Fleming Fund II GCP/GLO/710/UK “Engaging the food and agriculture sectors in sub-Saharan Africa and South and South-east Asia in the global efforts to combat antimicrobial resistance using a One Health approach”).

### Pilot Study Design

The Philippines is an archipelagic country located in Southeast Asia and consists of 7,641 islands. The country is divided into three major islands—Luzon, Visayas, and Mindanao. The study was conducted in representative provinces from the Luzon and Visayas group of islands, where the total population of swine and poultry is estimated at 12 and 197 million, respectively. Approximately 65 and 80% of the poultry and pigs, respectively, are raised in these two islands (7).

Ninety-three farms (four regions) across the Philippines voluntarily provided AMU information as part of a larger biosecurity and good practices study. Farms were enrolled with the assistance of a network of provincial veterinarians, extension service staff (LGUs), and regional AMR coordinators. Information sessions were held to discuss the study. The number of farms per region (Central Luzon, South Luzon, Central Visayas, and Western Visayas) was allocated based on their relative contribution to the national swine and poultry production. Within each province, farms were selected proportional to the species and production profiles. The categories of production systems were defined to classify farms into backyard farm operation (BYf) or commercial farm operation (CMf). For this study, BYf were defined as those having ≤500 layer or 1,000 broiler birds or ≤10 sows. On the other hand, CMf were defined as operations having ≥11 sows or ≥501 layer or 1,001 broiler birds. Depending on the province, researchers ensured that swine commercial grower operations varied in herd sizes to ensure representativeness.

Prior to participation in the interview, the researcher administered an informed consent form to the participating producer/designated farm staff. Interviews were conducted in English and Filipino. The questionnaire collected various pieces of information (please refer to Supplementary Materials I for additional details), but for the purposes of this study, reasons for AMU, AAIs, routes of administration, and stage of production where the AAIs were used (page 10 of the questionnaire) were extracted for analysis. Antimicrobials used pertain to the current cycle of broiler chickens, layers, and grower-finisher pigs, and other applicable stages, such as breeders, sows, and piglets for farms that have mixed production stages at the time of the study as indicated on page 3 of the questionnaire. Quantitative data on antimicrobial used on farm were not collected. The study was conducted from April to May 2018.

### Data Analysis

The data were entered in Microsoft Excel (Office 14) and analyzed descriptively using Microsoft Excel, Stata 15 (College Station, TX), and SAS version 9.4 (Cary, NC). The percentage of farms reporting using an antimicrobial over the total number of farms was the metric used to describe AMU. Proportions of the responses on AMU (i.e., the number of farms reporting use of each AAI) and AMU practices were compared between poultry and swine farms using either the Fisher exact test when there were five or fewer observations in any of the categories or the chi-square test in SAS 9.4. A *P*-value of ≤ 0.05 was considered significant, described as “significantly” or “statistically significant” throughout the text; actual *P-*values are specified in the tables. Comparisons of percentage of farms reporting use of each AAI between CMf and BYf were also made as detailed above. Reasons for use, categorized broadly by systems affected, the number of AAIs used by species, and production stage were analyzed descriptively. For this paper, the term “therapy” refers to both treatment and preventive uses. All AMU frequency and percentages information were organized by antimicrobial class.

## Results

The AMU data were voluntarily provided by a subset of farms (*n* = 93 farms) from 145 farms surveyed as part of the larger biosecurity study in the Philippines.

### Respondents and Farm Characteristics

**Figure 1:**
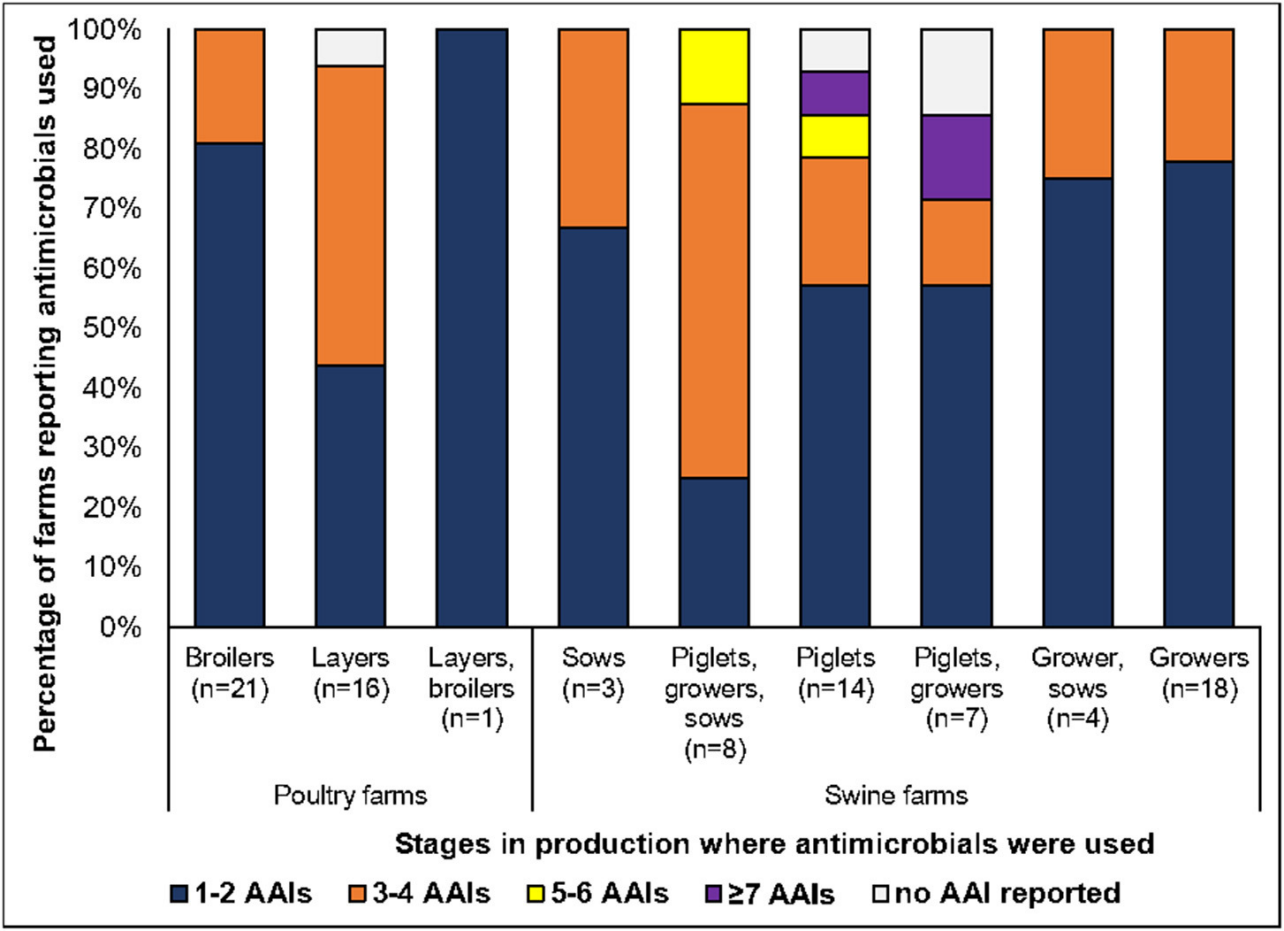
Percentage of farms using the total number of antimicrobials by poultry and swine and production stages. AAI, antimicrobial active ingredients. Respondents were asked to indicate the stage where they used the AAI (please refer to page 10 of Supplementary Materials I—Questionnaire). Several respondents have animals in their farm that comprised of more than one production stage.

The vast majority of the respondents were distributed between the age of 16 and 60 years (86%) and comprised of farm staff (16 CMf, 8% BYf), farm owners (11% CMf, 8% BYf), and veterinarians (11% CMf); the rest of the respondents did not specify their position or role in the rearing of animals. The 93 farms surveyed comprised of 35% (*n* = 33) BYf and 65% CMf (*n* = 60). By species, 43% (39 flocks) were poultry and the remaining 57% (54 herds) were swine. The 39 poultry farms in the study comprised of broiler flocks (*n* = 21), layers (*n* = 16), a broiler breeder, and a mixed layer–broiler farm. As summarized in Figure 1, the 54 swine farms comprised of single production stage herds (growers, piglets, sows) or mixed production stages present (e.g., mixed growers and piglets) in the farm at the time of the study. Most of the respondents in CMf (72%) indicated that their establishment has been operational for ≥ 5 years, whereas this proportion was smaller in BYf (43%). The majority of CMf (77%) had ≥ 3 barns, whereas most of BYf (75%) have 1 or 2 barns on their premises. Significantly higher percentage of CMf (70%) compared to BYf (11%) practiced all-in–all-out systems.

### AMU by Species

The spectrum of AAIs was more diverse in swine production (24 AAIs) compared to poultry (18 AAIs) (Table 1). Three AAIs were combination products (lincomycin-spectinomycin, trimethoprim-sulfamethoxazole, and trimethoprim-sulfadiazine). Significantly higher percentage of poultry farms used norfloxacin (poultry: 25% vs. swine: 6%). Erythromycin and fosfomycin were only used in poultry. In contrast, significantly higher percentage of swine farms used oxytetracycline (swine: 30% vs. poultry: 10%) and tylosin (swine: 25% vs. 8% poultry). Tiamulin and gentamicin were only used in swine. Enrofloxacin was the most frequently reported AAI in poultry and swine at 33% and 36%, respectively.

**Table 1:** Percentage of farms reporting the use of different antimicrobial active ingredients by animal species (in 39 poultry farms and 54 swine farms).

**Antimicrobial class**	**Antimicrobial active ingredient**	**Poultry farms *n* (%)**	**Swine farms *n* (%)**
Aminoglycosides	Apramycin	0 (0%)	1 (2%)
	**Gentamicin**	**0 (0%)**	7 (13%)^*^
	Neomycin	0 (0%)	1 (2%)
	Streptomycin	2 (5%)	2 (4%)
Cephalosporins	Ceftiofur	0 (0%)	2 (4%)
	Cephalexin	0 (0%)	1 (2%)
Fluoroquinolones	Ciprofloxacin	0 (0%)	1 (2%)
	Danofloxacin	0 (0%)	1 (2%)
	Enrofloxacin	13 (33%)	19 (36%)
	Levofloxacin	1 (3%)	0 (0%)
	**Norfloxacin**	**10 (25%)**	3 (6%)^*^
Lincosamides and aminocyclitols	Lincomycin	0 (0%)	3 (6%)
	Lincomycin-spectinomycin	0 (0%)	1 (2%)
Macrolides	**Erythromycin**	**3 (8%)**	0 (0%)^*^
	Kitasamycin	1 (3%)	0 (0%)
	Tilmicosin	2 (5%)	4 (8%)
	Tulathromycin	0 (0%)	1 (2%)
	**Tylosin**	**3 (8%)**	14 (25%)^*^
Penicillins	Amoxicillin	8 (20%)	11 (21%)
	Penicillin	1 (3%)	1 (2%)
Phenicols	Florfenicol	4 (10%)	5 (9%)
	Thiamphenicol	1 (3%)	0 (0%)
Phosphonic acid derivatives	**Fosfomycin**	**4 (10%)**	(0%)^*^
Pleuromutilins	**Tiamulin**	**0 (0%)**	12 (23%)^*^
Polypeptides	Colistin	5 (13%)	6 (11%)
Tetraycyclines	Chlortetracycline	0 (0%)	2 (4%)
	Doxycycline	6 (15%)	11 (21%)
	**Oxytetracycline**	**4 (10%)**	16 (30%)^*^
Trimethoprim and sulfonamides	Trimethoprim-sulfadiazine	5 (13%)	2 (4%)
	Trimethoprim-sulfamethoxazole	2 (5%)	0 (0%)

* *Significant differences between poultry and swine farms (P ≤ 0.05), Fisher exact test (represented in bold fonts)*.

### Number of Antimicrobials Used at Farm Level by Animal Production Stage and Reasons for Use

Figure 1 shows the percentage of farms using a different number of AAIs. The data were grouped according to species and the stage of production of the animals where the AAIs were used. One of the 93 farms was a broiler breeder farm that reported a non-antimicrobial feed additive (not shown in the figure). Forty-four percent to 81% of farms across all production categories used one to two antimicrobials. The use of 5 to 6 AAIs and ≥7 AAIs were observed in swine farms, mostly in piglet and piglet-grower herds (Figure 1).

The reported reason for AMU in poultry (Supplementary Materials II, **Annex 1**) was largely for respiratory diseases (17 AAIs) and a limited number (5 AAIs) were used for enteric diseases. Of note is the use of enrofloxacin (33%) and norfloxacin (25%) used for the therapy of respiratory diseases and colistin for both enteric and respiratory diseases. Similarly in swine, treatment of respiratory diseases was the most frequently indicated reason for use (20 AAIs). Enteric (17 AAIs), reproductive (4 AAIs), and non-specific (5 AAIs) diseases were additional reasons for use reported in swine. Of note is the use of enrofloxacin for the treatment of enteric, respiratory, and non-specific diseases.

### Route of Administration

Supplementary Materials II, **Annex 2** summarizes the AAIs by routes of administration; this varied by species depending on the AAI. In poultry, the vast majority of the respondents indicated that they administered the AAIs largely via water, whereas in swine, the most common route reported was intramuscular.

### AMU by Production System

Table 2 shows the percentage of farms from CMf and BYf production systems reporting specific AAIs. There were a total of 30 different AAIs belonging to 12 classes of antimicrobials documented. Overall, the spectrum of AAIs used among CMf was more diverse (29/30 AAIs) compared to those that were used in BYf (15/30 AAIs).

**Table 2:** Percentage of farms reporting the use of different antimicrobial active ingredients by production type (in 33 backyard farms and 60 commercial farms).

**Antimicrobial class**	**Antimicrobial active ingredient**	**Backyard farms n (%)**	**Commercial farms n (%)**
Aminoglycosides	Apramycin	1 (3%)	0 (0%)
	Gentamicin	2 (6%)	5 (8%)
	Neomycin	0 (0%)	1 (2%)
	Streptomycin	2 (6%)	2 (3%)
Cephalosporins	Ceftiofur	0 (0%)	2 (3%)
	Cephalexin	0 (0%)	1 (2%)
Fluoroquinolones	Ciprofloxacin	0 (0%)	1 (2%)
	Danofloxacin	0 (0%)	1 (2%)
	Enrofloxacin	8 (24%)	24 (40%)
	Levofloxacin	0 (0%)	1 (2%)
	**Norfloxacin**	**0 (0%)**	13 (22%)^*^
Lincosamides and aminocyclitols	Lincomycin	0 (0%)	3 (5%)
	Lincomycin-spectinomycin	0 (0%)	1 (2%)
Macrolides	Erythromycin	0 (0%)	3 (5%)
	Kitasamycin	0 (0%)	1 (2%)
	Tilmicosin	1 (3%)	5 (8%)
	Tulathromycin	0 (0%)	1 (2%)
	Tylosin	6 (18%)	10 (17%)
Penicillins	**Amoxicillin**	**1 (3%)**	18 (30%)^*^
	Penicillin	1 (3%)	1 (2%)
Phenicols	Florfenicol	1 (3%)	8 (13%)
	Thiamphenicol	0 (0%)	1 (2%)
Phosphonic acid derivatives	Fosfomycin	0 (0%)	4 (7%)
Pleuromutilins	Tiamulin	2 (6%)	10 (17%)
Polypeptides	**Colistin**	**1 (3%)**	10 (17%)^*^
Tetracyclines	Chlortetracycline	1 (3%)	1 (2%)
	Doxycycline	5 (15%)	12 (20%)
	**Oxytetracycline**	**13 (39%)**	7 (12%)^*^
Trimethoprim and sulfonamides	Trimetoprim-sulfadiazine	3 (9%)	4 (7%)
	Trimetoprim-sulfamethoxazole	0 (0%)	2 (3%)

* *Significant differences between backyard and commercial farms (P ≤ 0.05), Fisher exact test (represented in bold fonts)*.

A significantly higher percentage of CMf reported use of amoxicillin and colistin (30% and 22% CMf vs. 3% BYf, respectively). Some AAIs such as norfloxacin were reported only in CMf. A significantly higher proportion of BYf used oxytetracycline (39% BYf vs. 12% CMf).

### Access to Antimicrobials, Sources of Advice, and Related AMU Practices

When respondents were asked about the frequency of use, a vast majority indicated that they treat their animals only when the animals were sick or showed clinical signs (73% CMf and 83% BYf). In terms of access to antimicrobials, significantly higher proportion of BYf (30%) compared to CMf (9%) accessed AAIs over-the-counter from agrovet supply or retail outlets. Agrovet supply or retail outlets are local stores that typically sell VMPs, livestock, and farm equipment and supplies. CMf accessed antimicrobials largely from their integrator/company that supplied them with other farm inputs or directly from pharmaceutical companies (18%). A relatively small proportion of farms obtained VMPs with veterinary prescription from agrovet supply or retail outlets (6% BYf, 11% CMf).

For informed AMU decisions, a significantly higher percentage of CMf consulted with veterinarians (43% CMf vs. 18% BYf), whereas BYf more often consulted with paraveterinarians (28% BYf vs. 2% CMf). The rest of the BYf and CMf obtained advice from drug company representatives and relied on their own farm experiences in treating their animals and on the advice from agrovet supply staff or other producers.

Responses to the general reasons for using antimicrobials were relatively similar between the BYf and CMf where there was a relatively equal distribution of prevention or treatment alone, both prevention and treatment, and prevention, treatment, and growth promotion. In the event that the flocks or herds were unresponsive to antimicrobial therapy, a significantly higher proportion of CMf conducted necropsy (63% CMf vs. 21% BYf) or euthanasia followed by disposal of dead animals in designated sites within the farm (30% CMf vs. 13% BYf), whereas BYf took no action (40% BYf vs. 7% CMf).

## Discussion

This qualitative pilot study provides an overview of the AAIs used in poultry and swine CMf and BYf in the Philippines, the reasons why AAIs are used, and common AMU practices, including how producers access AAIs and whom they consult for AMU advice. Increasing demand for chickens and pork and the potential public health implications of the consumption of these products contaminated with antimicrobial resistant foodborne pathogens (12–16) emphasized that AMU surveillance in these food animals should be prioritized.

In this study, we used a simple count-based measurement indicating percentage of farms reporting the use of certain AAIs. Count-based measurements of AMU at the farm level such as the number of days and the number of medicated rations or water treatments and injections are commonly used as numerators in less sophisticated AMU surveillance programs (23). These measurements are useful to compare percentages of AAIs by species and between farms over time, to describe seasonal variations of use or shifts in AMU options (5), and to monitor the progress of interventions to reduce AMR (6). Our metric detected variations in the spectrum of antimicrobials used, the number of AAIs used in poultry and swine and in relevant production stages, and between BYf and CMf. An important finding is the use in poultry and swine CMfs of AAIs belonging to fluoroquinolones and polypeptides, classes categorized by the World Health Organization (WHO) as highest priority critically important antimicrobials (CIAs), and phosphonic acid derivatives, categorized as a high-priority CIA (24). Though at lower percentages, BYfs reportedly used the same classes. The spectrum of AAIs in our study is comparable to other LMICs in Southeast Asia with similar livestock farming systems (CMf and BYf) such as Indonesia, Vietnam, and Thailand (25, 26). The use of these AAIs is consistent with the detection of *E. coli* resistant to cephalosporins from poultry (13) and swine (12, 15, 16) and the detection of ciprofloxacin resistant *Campylobacter* from retail chickens (14) sampled within the same regions in our study. As evidenced by the relatively common practice of over-the-counter purchase of VMPs from agrovet shops or retail outlets (largely by BYf), enhanced veterinary oversight or VCPR and regulating access to these antimicrobials such as prescription-only use are required. This may involve monitoring the off-label use or restrictions on the metaphylactic use of antimicrobials belonging to WHO's Essential List of Medicines such as colistin (27).

Overall, respiratory disease was the most commonly reported reason for use in poultry and swine farms. The use of antimicrobials for enteric diseases was more common among swine farms, particularly in herds that comprised of piglets and growers. A proportion of swine producers indicated that they used AAIs, but the diseases they treated were not specified, emphasizing that the diagnosis and clinical assessments of the flock/herd conditions for informed AMU decisions need to be improved. These findings indicate that next to AMU data, more detailed information of diseases driving AMU in poultry and swine in the Philippines is needed to inform guidelines on prudent AMU and other interventions to curb AMU including refinements of vaccination and other preventive health programs.

From a surveillance standpoint, our study has certain limitations including the collection of more comprehensive data to enable quantitative estimation of farm-level AMU (23, 28, 29). However, our study provided a descriptive landscape of AMU practices (between production systems and stages of production). Commercial farms and BYf production systems both contribute to the national demand for poultry and swine products in the Philippines. The latter production system is relatively common as these farms are essential for food security in LMICs such as the Philippines for supplying local and remote areas and source of livelihood. The potential contribution of these production systems to the overall AMU quantity and food safety implications makes indispensable their inclusion in a national AMU surveillance program. Furthermore, the survey indicated that some farms constituted of mixed production stages (piglets/grower/sows, piglets/growers, layer/broiler), which may add complexity to a national AMU data collection, but the framework could target those stages that are closest to the consumer such as broiler chickens, layers, and growers, being more reflective of the potential AMR in foodborne pathogens from the meat and egg products. Our data also showed that diverse antimicrobials (up to seven AAIs) involved herds that contain young animals such as piglets, suggesting that this production stage should also be included in a national farm-level AMU surveillance for informing interventions to address health issues in young animals. Because national farm-level AMU surveillance would require human resources and ongoing national funding for operationalizing the farm data collection, future farm surveillance design may explore inexpensive farm-level AMU methodology, such as “garbage can audit” (30).

Our findings emphasized the urgent need for curbing the use of CIAs in poultry and swine farms in the Philippines and the need for changes in antimicrobial VMP regulations that pertain to their dispensation, in particular, where BYf frequently access these products (agrovet shops and retail outlets and their distributors). Enhanced veterinary oversight and ongoing national AMU monitoring will inform interventions to offset the need for AMU and reduction of AMR risks arising from food animals in the Philippines.

## Data Availability Statement

All datasets generated for this study are included in the article/Supplementary Material.

## Ethics Statement

This farm-level survey involving producers is part of a larger study (good practices survey as part of the nationwide antimicrobial resistance [AMR] campaign in the Philippines IAMResponsible) and was reviewed and approved by the Bureau of Animal Industry-AMR program designates and the Regional AMR Coordinators. The participants provided their written informed consent to participate in this study.

## Author Contributions

TB, RM, and CB conceived the study and developed the questionnaires and the sampling frame. TB, RM, MMC, and MBC contributed to the coordination of workshops, meetings with industry, and other stakeholders (industry and veterinary associations), the regional networks (veterinarians and staff from the local government units), and the recruitment of participants. TB, AA, and AD-G contributed to data validation and analysis. KB provided supervision and overall technical guidance to the project. All authors contributed to the writing and editing of the manuscript. All authors contributed to the article and approved the submitted version.

## Conflict of Interest

The authors declare that the research was conducted in the absence of any commercial or financial relationships that could be construed as a potential conflict of interest.

## Supplementary Material

Supplementary Materials I Questionnaire: informed consent form and farm questionnaire on antimicrobial use (page 10), good husbandry practices and biosecurity.

Supplementary Materials II Other results: reasons for antimicrobial use and routes of administration in poultry and swine commercial and backyard farms in the Philippines.
