# Supplementary material for: Antimicrobials Used in Backyard and Commercial Poultry and Swine Farms in the Philippines: A Qualitative Pilot Study
Source: Front Vet Sci. 2020 Jul 8;7:329. doi: 10.3389/fvets.2020.00329 (PMC7360799; doi:10.3389/fvets.2020.00329)
Supplement: Supplementary Materials I — Questionnaire: informed consent form and farm questionnaire on antimicrobial use (page 10), good husbandry practices and biosecurity. [file Data_Sheet_1.PDF]

Survey on Antimicrobial Use in Swine and Poultry Production Systems in the Philippines

Dear Respondent,

Thank you for taking time to participate in this survey.

As part of the ongoing efforts of the government in creating evidence-based policies regarding Antimicrobial Resistance (AMR), we are conducting a survey to document farm management practices, including antimicrobial use in different swine and poultry production systems in the Philippines. Because you are engaged in farm operations, we would like to seek assistance from you by completing the attached questionnaire.

This questionnaire will require approximately 15 minutes to complete. Kindly answer all the questions honestly based on your current farm practices and return the completed forms to the researcher. Rest assured that all your answers will be treated with utmost confidentiality.

Sincerely,

BAI-FAO Antimicrobial Resistance Project
Bureau of Animal Industry
Visayas Avenue, Diliman, Quezon City
Philippines

Consent Form on Antimicrobial Use in Swine and Poultry Production Systems Survey in the Philippines

Direction: Kindly place a check mark in the boxes on the right side of every statement

1. I understand that my participation in the said survey is voluntary and I can withdraw anytime during the interview. ☐
2. I confirm that I have read, understood and answered all the question in this survey which is deemed relevant in the creation of their baseline data on antimicrobial use in swine and poultry farms in the Philippines ☐
3. I agree to take part in this study and also understand that my identity and all my answers will be protected. ☐

Signed by:

______________________	______________________	______________________
**Name of Respondent**	**Date Accomplished**	**Signature**

Survey on Antimicrobial Use in Swine and Poultry Production Systems in the Philippines

A. Personal Profile

**Name of Respondent**	
**Age**	( ) 16-30 ( ) 31-45 ( ) 46-60 ( ) 61 &above
**Gender**	( ) Male ( ) Female
**Educational Attainment**	( ) Primary ( ) Secondary ( )Vocational ( )Tertiary
**Position in the Farm**	
**Formal Education in Animal Production System**	( ) Yes ( ) No ( ) If yes, what? ____________

B. Farm Profile

**Name of Farm**	
**Address**	
**GPS Coordinates**	Longitude: Latitude:
**Age of Farm**	( ) < 1 yr. ( ) 1-2 yrs. ( ) 3-4 yrs. ( ) 5 & above
**Number of Houses per Farm**	( ) 1 ( ) 2 ( ) 3 ( ) 4 & above
**Major Species Present**	( ) Poultry ( ) Swine
**Other species present, please specify**	
**Type of Poultry***	( ) Broiler ( ) Layer ( ) Breeder
**Type of Swine***	( ) Farrow to Finish ( ) Farrow to Feeder<br>( ) Feeder to Finish
**Type of Production System**	( ) Backyard ( ) Commercial
**If swine commercial, please classify further**	( ) Small ( ) Medium ( ) Large

**Please check all applicable for your farm*

Classification of Production System

1. Poultry
   - Backyard[1] – ≤ 500 layers, 1,000 broiler birds
   - Commercial[2] – ≥ 501 layers or ≥1,001 broilers
2. Swine
   - Backyard – ≤10 sows
   - Commercial
     - Small – ≥ 11 sows
     - Medium – 100-300 sows
     - Large – more than 300 sows

[1] **Backyard** – small flock or herd and your production is intended for the consumption of the general public in your area or nearby municipalities; if you are raising free-ranging birds intended for household consumption, please do not answer the questionnaire.
[2] Commercial – you are under an integrated system (company supplies producton such as animals, vaccines and veterinary drugs, technical service) or an independent producer (you source your own animals, feeds, vaccines and veterinary drugs and technical service).

Direction: Please answer this questionnaire by writing a (✓) based on your farm management practices. *Write n/a or don't know if not applicable for your farm or you do not know the answer.*

C.1	Housing	
1.1	Is the farm located near the residential area?	( ) Yes ( ) No
1.2	What is the enclosure surrounding your farm? *Please check all applicable.*	( ) fence<br>( ) concrete walls<br>( ) natural vegetation<br>( ) none
1.3	What are the sources of ventilation in your farm? *Please check all applicable.*	( ) electric fans<br>( ) blower or industrial fans<br>( ) wet pad cooling system<br>( ) natural air
1.4	How do you keep the animals in the farm? *Please check all applicable.*	( ) fence of any material (bamboo, wood)<br>( ) concrete housing pens<br>( ) free range
1.5	Describe the accessibility of farms to roads. *Please check all applicable*	( ) Farm is near the market<br>( ) Farm is 5 kilometers away from the nearest highway and accessed through a cemented road<br>( ) Farm is located in an isolated area and takes more than 5 kilometers before reaching the nearest highway and accessed through a non-cemented roads
**C.2**	**Source of Animals**	
2.1	How did you acquire animals in the farm? *Please check all applicable or specify:*	( ) integrators through contract growing<br>( ) swine breeder farm<br>( ) poultry breeder farms<br>( ) Others, please specify:<br>____________________
2.2	What do you do when new animals are scheduled to arrive/arrive at the farm? *Please check all applicable*	( ) Clean and disinfect the farm house before arrival of new animals<br>( ) Disinfect the farm house<br>( ) Check health and warranty records of the animals<br>( ) Quarantine or isolate animals for a given period<br>( ) Give water.<br>( ) Feed tha animals<br>( ) Others, please specify:<br>____________________
**C.3**	**Feed and Nutrition**	
3.1	Where do you purchase feeds of animals?	( ) Integrators<br>( ) Agri-vet supply store<br>( ) Own feed formulation<br>( ) Others, please specify:<br>____________________
3.2	If feeds are formulated by the farm, how are these mixed? *Please check all applicable*	( ) The farm's feed formulation is mixed by own feed mill<br>( ) Hiring of toll manufacturer to mix feeds for the farm based on our own feed formulation<br>( ) Mix my own feeds depending on what is available<br>( ) Others, please specify:<br>____________________
3.3	How do you store your feeds or feed ingredients in the farm?	( ) Feed storage room<br>( ) Beside the cage/pen<br>( ) Others, please specify:<br>____________________

3.4	How often do you provide feeds to the animals per day?	( ) once ( ) twice<br>( ) thrice ( ) ad libitum
3.5	What is your feeds inventory system?	( ) First in, First out (first feed delivered, first feed to be consumed)<br>( ) Last in, First out (last feed delivered, first feed to be consumed)<br>( ) No inventory management
3.6	What additives do you incorporate/do you think are incorporated in your feeds? *Please check all applicable and provide specific additives.*	( ) Vitamins such as ______________________<br>( ) Minerals such as ______________________<br>( ) Antibiotics such as ______________________<br>( ) None
**.C.4**	**Water**	
4.1	Do you give water to the animals?	( ) Yes ( ) No
4.2	What is the water source of the farm?	( ) deep well<br>( ) commercial pump<br>( ) water pump
4.3	Are there any microbiological tests done in your farms to test water potability?	( ) Yes ( ) No
4.4	If you answer YES to 4.3, how often are the water tests conducted?	( ) once/month<br>( ) quarterly<br>( ) twice/year
4.5	Do you treat water in the farm?	( ) Yes ( ) No
4.6	If you answer YES to 4.5, how do you treat the water in the farm?	( ) By putting chlorine<br>( ) By exposing it to ultraviolet treatment<br>( ) By adding lime or water ash<br>( ) Others, please specify: ______________________
4.7	If you answer YES to 4.5, how often do you treat the water?	( ) once/month<br>( ) quarterly<br>( ) twice/year
**C.5**	**Biosecurity**	
5.1	How do vehicles enter your farm premises?	( ) Vehicles need to dip on a tire bath<br>( ) Vehicle tires are sprayed with disinfectants<br>( ) Vehicles readily enter the farm premises and stop at the parking lot<br>( ) Others, please specify: ______________________
5.2	What do you do when people enter your farm premises? *Please check all applicable*.	( ) **Visitors need to step on the foot bath for disinfection**<br>( ) Visitors readily enter the farm<br>( ) The farm has a log book for people to log in before entering the farm<br>( ) People entering the farm are required to change to working clothes and footwear provided by the farm<br>( ) Changing to working clothes and footwear are not required<br>( ) People need to shower before and after entering the farm<br>**( ) Taking a shower before and after entering the farm are not required**<br>( ) Handwashing(with soap) before and after entering the farm is required.<br>( ) Handwashing before and after entering the farm is not required.
5.3	If you use disinfectants when entering the farm, please answer questions 5.3-5.6. How	( ) everyday<br>( ) twice/week

	often do you change disinfectants in the foot and tire bath?	( ) every week
5.4	Do you change the type of disinfectants (quaternary ammonium, glutaraldehyde, formaldehyde, iodine, phenols, chlorine, etc.) in your farm?	( ) Yes ( ) No
5.5	If you answer YES to 5.4, how often do you change the type of disinfectants?	( ) once/month<br>( ) quarterly<br>( ) twice/year
5.6	Do you practice “all-in, all-out” of animals in your farm operations?	( ) Yes ( ) No<br>( ) Not applicable
5.7	Do you have any pest (flies, mosquitoes, etc.) control measures in the farm?	( ) Yes ( ) No
5.8	Do you have a mortality pit within the farm premises where you bury dead animals?	( ) Yes ( ) No
5.9	If you answer NO to 5.8, where do you dispose dead animals?	( ) incineration<br>( ) fed to other animals<br>( ) processed as fertilizer<br>( ) others, please specify:<br>______________________
5.10	Is your farm registered or accredited by any government or non-government program?	( ) Yes ( ) No
5.11	If you answer YES to 5.10, what is/are this/these accreditation program/s?	( ) Good Animal Husbandry Practices<br>( ) Animal Welfare Act<br>( ) Swine Breeder Accreditation Program<br>( ) Avian Influenza Free Farm<br>( ) Others, please specify:<br>_______________________
**C.6**	**Record Keeping**	
6.1	Do you keep records in the farm?	( ) Yes ( ) No
6.2	If you answer YES to 6.1, please answer to 6.2 to 6.6. How do you keep farm records?	( ) use of log book/notebook<br>( ) computer-based record management<br>( ) others, please specify:<br>______________________
6.3	Do you keep a written record on the population of animals in the farm?	( ) Yes ( ) No
6.4	Do you keep a written record of the whole production cycle of your animals? (example: how many broilers, ready to lay,etc.)	( ) Yes ( ) No<br><br>( ) certain stage only (during _____)
6.5	How often do you record the population of animals?	( ) daily<br>( ) weekly<br>( ) monthly
6.6	Describe your usual recording practices. Check as applicable.	( ) The farm has a written record on all the animals purchased by the farm<br>( ) The farm has a written record on all the animals to be slaughtered or sold at any given time<br>( ) The farm has a written record on the different performance parameters (weight, feed intake, mortality etc)<br>( ) The farm has a written record for treated animals with clinical signs<br>( ) The farm has a written record on all medications purchased in the farm<br>( ) All antibiotics/antimicrobials administered to the animals in the farm are recorded<br>( ) The farm has a written record on all the movement of animals within the farm

C.7	Animal Health Management	
7.1	Describe your animal health management practices in the farm. *Please check all applicable*.	( ) We consult a veterinarian when we observe sick animals in the farm<br>( ) We consult veterinary paraprofessional, livestock inspector or agricultural technician when we observe sick animals in the farm<br>( ) We rely on friend's advice in treatment of sick animals<br>( ) We rely on personal farm experience in treatment of animals<br>( ) We isolate sick animals on a separate pen/place<br>( ) We administer vaccines to prevent entry of diseases in the farm<br>( ) We administer different antibiotics to treat and prevent diseases<br>( ) We rely on herbal medicines to treat sick animals<br>( ) Others, please specify:<br>____________________
7.2	What practices will prevent diseases in the farm? Please rank your answers with one as the most important.	( ) implement a vaccination program<br>( ) clean and disinfect the houses/pens regularly<br>( ) administer minerals and vitamins in the feeds<br>( ) administer antibiotics in the feeds always<br>( ) administer antibiotics only when needed<br>( ) set up measures to control movement of people to the farm<br>( ) set up measures to control movement of vehicles to the farm
7.3	Are you familiar with antibiotics/antimicrobials?	( ) Yes ( ) No
7.4	If you answer YES to 7.3, please answer 7.4 to 7.5. Which of the following choices best describes antimicrobials used in animals.	( ) These are drugs which kills or inhibits the growth of different microorganisms such as bacteria, virus, fungi or protozoa but has little effect or no damage to the animals<br>( ) These are drugs used to kill different microorganisms in animals<br>( ) These are drugs used to inhibit different microorganisms in animals<br>( ) I don't know
7.5	Which of the following choices best describes antibiotics used in animals.	( ) This is a type of antimicrobial that kills or inhibits the growth of bacteria<br>( ) This is a type of antimicrobial that kills different microorganisms such as bacteria, virus, fungi or protozoa<br>( ) Antibiotics and antimicrobials have the same definition<br>( ) I don't know
7.6	Do you use any antibiotics/antimicrobials in your farm?	( ) Yes ( ) No
7.7	If you answer YES to 7.6, what are the usual types of antibiotics/antimicrobials used in your farm?	( ) Anti-bacterial<br>( ) Anti-viral<br>( ) Anti-parasitic<br>( ) Don't know
7.8	Other than antibiotics/antimicrobials, do you administer any supportive medication in your farms?	( ) Yes ( ) No

7.9	If you answer YES to 7.8, what is/are this/these supportive medication? Check as applicable	( ) Vitamins/Minerals<br>( ) Electrolytes<br>( ) Probiotic/Prebiotics<br>( ) Enzymes<br>( ) Acidifier<br>( ) Betaglucans/Mannanoligosaccharides<br>( ) Others, please specify:<br>____________________
7.10	If you answer YES to 7.6, please answer 7.10 to 7.22. How many antibiotics/antimicrobials types/kinds do you use in your farm operations?	( ) None<br>( ) 1-2<br>( ) 3-4<br>( ) 5-6<br>( ) 6 or more<br>( ) don't know
7.11	Where do you store antibiotics/antimicrobials in the farm?	( ) refrigerator<br>( ) storage room<br>( ) inside piggery and poultry house<br>( ) Others, please specify:<br>____________________
7.12	Do you practice "first-in, first-out" (first purchased, first to be given) in administration of antibiotics/antimicrobials to animals?	( ) Yes ( ) No
7.13	How frequent do you use antibiotics/antimicrobials in your medication program?	( ) All the time<br>( ) Once a week<br>( ) Twice a month<br>( ) Only when they are sick<br>( ) Others, please specify:<br>_________________
7.14	When do you administer antibiotics/antimicrobials to the animals?	( ) When new animals are introduced (day of arrival or after giving birth)<br>( ) When animals show signs of weakness or lameness<br>( ) When animals are apparently healthy<br>( ) When instructed by veterinarian<br>( ) Others
7.15	How are antibiotics/anitimicrobials admininistered to your animals in the farm? *Please check all applicable.*	( ) in-water<br>( ) in-feed<br>( ) injectable/parenteral<br>( ) topical
7.16	How do you acquire antibiotics/antimicrobials for your farm operations?	( ) purchased through agri-vet supply stores<br>( ) purchased through agri-vet supply stores with veterinary prescription<br>( ) provided by veterinarians or veterinary paraprofessionals (livestock inspector, agricultural technicians)<br>( ) provided by integrators in contract growing businesses<br>( ) provided by veterinary drug companies
7.17	What is your purpose of using antibiotics/antimicrobials? *Please check all applicable*.	( ) To prevent disease (prophylactic)<br>( ) To treat disease (therapeutic)<br>( ) To prevent and treat disease (prophylactic and therapeutic)<br>( ) To promote growth (growth promotion)

		( ) To prevent and treat disease, promote growth (prophylactic, therapeutic, growth promotion)<br>( ) To minimize stress conditions
7.18	What is your basis for choosing antibiotics/antimicrobials ?	( ) veterinarian's advice<br>( ) veterinary paraprofessionals'advice (livestock inspector, agricultural technicians)<br>( ) pharmaceutical representatives<br>( ) farm history experience<br>( ) recommendation of seller from agri-vet supply stores<br>( ) Others (social media, online articles, friends other farm practitioners)
7.19	If there are sick animals which did not recover from antibiotics/antimicrobials, what do you do?	( ) wait until the animal naturally dies<br>( ) sacrifice the animal and bury it<br>( ) immediately sell the animals<br>( ) subject the animal for necropsy to diagnose disease present<br>( ) Others, please specify:<br>___________________
7.20	Do you know the term "withdrawal time" in farming practices?	( ) Yes ( ) No
7.21	If you answer YES to 7.20, do you practice withdrawal time when administering antibiotics/antimicrobials in animals that are about to be sold to the market or "*viajeros*"[3]	( ) Yes ( ) No
7.22	If you answer YES to 7.21, where do you seek advice on proper antibiotics/antimicrobials withdrawal time?	( ) Veterinarian<br>( ) Farm history experience<br>( ) Pharmaceutical Representative<br>( ) Product Label<br>( ) Others (social media, online articles, other farm practitioners)

[3] These are independent buyers of live animals and have their own marketing networks (processors, wet markets, butcher shops).

Common Disease Problem/s	Drug of Choice/s	Route of Administration (in-feed, in-water, intramuscular, subcutaneous, topical)	Indicate at what stage where they used Poultry (layer, breeder, broiler) Swine (piglet, grower, sow)
