# Supplementary material for: Antimicrobials Used in Backyard and Commercial Poultry and Swine Farms in the Philippines: A Qualitative Pilot Study
Source: Front Vet Sci. 2020 Jul 8;7:329. doi: 10.3389/fvets.2020.00329 (PMC7360799; doi:10.3389/fvets.2020.00329)
Supplement: Supplementary Materials II — Other results: reasons for antimicrobial use and routes of administration in poultry and swine commercial and backyard farms in the Philippines. [file Data_Sheet_2.pdf]

SUPPLEMENTARY MATERIALS – PART II

ANNEX 1 | Reported reasons for antimicrobial use by systemic syndromes/diseases in poultry and swine commercial and backyard farms in the Philippines

A. Poultry (n = 39 farms).

Antimicrobial class/active ingredient	Enteric n (%)	Respiratory n (%)[1]
**Aminoglycosides**		
Streptomycin	-	2 (5%)
**Fluoroquinolones**		
Enrofloxacin	-	13 (33%)
Levofloxacin	-	1 (3%)
Norfloxacin	-	10 (26%)
**Macrolides**		
Erythromycin	-	3 (8%)
Kitasamycin	-	1 (3%)
Tilmicosin	-	2 (5%)
Tylosin	-	3 (8%)
**Penicillin**		
Amoxicillin	2 (5%)	8 (20%)
Penicillin	-	1 (3%)
**Phenicols**		
Florfenicol	-	4 (10%)
Thiamphenicol	-	1 (3%)
**Phosphonic acid derivatives**		
Fosfomycin	-	4 (10%)
**Polypeptides**		
Colistin	2 (5%)	3 (8%)
**Tetracyclines**		
Doxycycline	1 (3%)	5 (13%)
Oxytetracycline	-	4 (10%)
**Trimethoprim and sulfonamides**		
Trimethoprim-sulfadiazine	1 (3%)	4 (10%)
Trimethoprim-sulfamethoxazole	2 (5%)	-

[1] Percentage is the number of farms using the antimicrobial divided by the total number of poultry farms; please note that responses were not mutually exclusive where one farm may have used the same antimicrobial for the treatment of both enteric and respiratory diseases.
- no response for the antimicrobial and reason for use.

B. Swine (n = 54 farms).

Antimicrobial class/active ingredient	Enteric n (%)[1]	Reproductive n (%)	Respiratory n (%)	Nonspecific n (%)
Aminoglycosides				
Apramycin	1 (2%)	-	-	-
Gentamicin	1 (2%)	3 (6%)	3 (6%)	-
Neomycin	1 (2%)	-	-	-
Streptomycin	1 (2%)	-	1 (2%)	-
Cephalosporins				
Ceftiofur	-	-	2 (4%)	-
Cephalexin	1 (2%)	-	-	-
Fluoroquinolones				
Ciprofloxacin	1 (2%)	-	-	-
Danofloxacin	-	-	-	1 (2%)
Enofloxacin	9 (17%)	-	11 (21%)	1 (2%)
Norfloxacin	-	-	3 (6%)	-
Lincosamides				
Lincomycin	1 (2%)	-	2 (4%)	-
Lincomycin-spectinomycin	1 (2%)	-	1 (2%)	-
Macrolides				
Tilmicosin	-	-	4 (8%)	-
Tulathromycin	-	-	1 (2%)	-
Tylosin	5 (9%)	-	7 (13%)	-
Penicillin				
Amoxicillin	1 (2%)	1 (2%)	8 (15%)	1 (2%)
Penicillin	-	-	1 (2%)	
Phenicols				
Florfenicol	1 (2%)	-	6 (11%)	-
Pleuromutilins		-	8 (15%)	-
Tiamulin	4 (8%)	-	8 (15%)	-
Polypeptides				
Colistin	2 (4%)	-	4 (8%)	-
Tetracycline				
Chlortetracycline	2 (4%)	-	1 (2%)	-
Doxycycline	2 (4%)	1 (2%)	7 (13%)	1 (2%)
Oxytetracycline	7 (13%)	4 (8%)	8 (15%)	2 (4%)
Trimethoprim and sulfonamides				
Trimethoprim-sulfadiazine	-	-	2 (4%)	-

[1] Percentage is the number of farms divided by the total number of swine farms; please note that responses were not mutually exclusive where one farm may have used the same antimicrobial for various reasons.
- no response for the antimicrobial and reason for use.

ANNEX 2 | Reported routes of administration of the antimicrobials used on farm

A. Routes of administration in poultry (n=39 farms).

Antimicrobial class/active ingredient	Feed n (%)[1]	Intramuscular n (%)	Water n (%)	Both water and intramuscular n (%)[2]
**Aminoglycosides**				
Streptomycin	-	-	2 (5%)	-
**Fluoroquinolones**				
Enrofloxacin	-	-	13 (33%)	1 (3%)
Levofloxacin	-	-	1 (3%)	-
Norfloxacin	-	-	9 (23%)	1 (3%)
**Phosphonic acid derivatives**				
Fosfomycin	-	-	4 (10%)	-
**Macrolides**				
Erythromycin	-	-	3 (8%)	-
Kitasamycin	-	-	1 (3%)	-
Tilmicosin	-	-	2 (5%)	-
Tylosin	-	-	3 (8%)	-
**Penicillins**				
Amoxicillin	1 (3%)	-	8 (20%)	-
Penicillin	-	-	1 (3%)	-
**Phenicols**				
Florfenicol	-	-	4 (10%)	-
Thiamphenicol	-	-	1 (3%)	-
**Polypeptides**				
Colistin	-	-	5 (13%)	-
Tetracycline				
Doxycycline	-	-	6 (15%)	-
Oxytetracycline	-	1 (3%)	3 (8%)	-
**Trimethoprim and sulfonamides**				
Trimethoprim-sulfadiazine	-	1 (3)	4 (10%)	-
Trimethoprim-sulfamethoxazole	-	-	2 (5%)	-

[1] Percentage is the number of responses divided by the total number of poultry farms; please note that responses were not mutually exclusive where one farm may have used the same antimicrobial administered via different routes of administrations.
[2] These antimicrobial active ingredients have pharmaceutical forms intended for both oral and intramuscular administration.
- no response for the antimicrobial and reason for use.

B. Routes of administration in swine (n=53 farms).

Antimicrobials class/active ingredient	Feed n (%)	Both feed and intramuscular n (%)	Intramuscular n (%)	Water n (%)
**Aminoglycosides**				
Apramycin	-	-	-	1 (2%)
Gentamicin	-	-	7 (13%)	-
Neomycin	1 (2%)	-	-	-
Streptomycin	-	-	-	2 (4%)
**Cephalosporins**				
Ceftiofur	-	-	2 (4 %)	
Cephalexin	-	-	-	1 (2%)
**Fluoroquinolones**				
Ciprofloxacin	-	-	-	1 (2%)
Danofloxacin	-	-	1 (2%)	-
Enrofloxacin	-	1 (2%)	18 (34%)	2 (4%)
Norfloxacin	-	-	2 (4%)	1 (2%)
**Lincosamides and aminocyclitols**				
Lincomycin	-	1 (2%)	2 (4%)	-
Lincomycin-spectinomycin	1 (2%)	-	1 (2%)	-
**Macrolides**				
Tilmicosin	2 (4%)	1 (2%)	1 (2%)	-
Tulathromycin	-	-	1 (2%)	-
Tylosin	1 (2%)	1 (2%)	9 (17%)	1 (2%)
**Penicillin**				
Amoxicillin	-	3 (6%)	7 (13%)	1 (2%)
Penicillin	-	-	1 (2%)	-
**Phenicols**				
Florfenicol	-	4 (8%)	3 (6%)	-
**Pleuromutilins**				
Tiamulin	4 (8%)	3 (6%)	4 (8%)	1 (2%)
**Polypeptides**				
Colistin	1 (2%)	1 (2%)	-	4 (8%)
**Tetracyclines**				
Chlortetracycline	2 (4%)	-	1 (2%)	-
Doxycycline	2 (4%)	3 (6%)	2 (4%)	4 (8%)
Oxytetracycline	1 (2%)	1 (2%)	19 (36%)	-
**Trimethoprim and sulfonamides**				
Trimethoprim-sulfadiazine	-	-	2 (4%)	1 (2%)

[1] Percentage is the number of responses divided by the total number of swine farms; please note that responses were not mutually exclusive where one farm may have used the same antimicrobial administered via different routes of administrations.

[2]*These antimicrobial active ingredients have pharmaceutical forms intended for both oral and intramuscular administration.*

- no response for the antimicrobial and reason for use.
